# Unambiguous State Discrimination with Intrinsic Coherence

**DOI:** 10.3390/e24010018

**Published:** 2021-12-23

**Authors:** Jin-Hua Zhang, Fu-Lin Zhang, Zhi-Xi Wang, Hui Yang, Shao-Ming Fei

**Affiliations:** 1 Department of Physics, Xinzhou Teacher’s University, Xinzhou 034000, China; yanghui20032002@163.com; 2 Department of Physics, School of Science, Tianjin University, Tianjin 300072, China; 3 School of Mathematical Sciences, Capital Normal University, Beijing 100048, China; wangzhx@mail.cnu.edu.cn; 4 Max-Planck-Institute for Mathematics in the Sciences, D-04103 Leipzig, Germany

**Keywords:** mixed state discrimination, coherence, quantum filtering

## Abstract

We investigate the discrimination of pure-mixed (quantum filtering) and mixed-mixed states and compare their optimal success probability with the one for discriminating other pairs of pure states superposed by the vectors included in the mixed states. We prove that under the equal-fidelity condition, the pure-pure state discrimination scheme is superior to the pure-mixed (mixed-mixed) one. With respect to quantum filtering, the coherence exists only in one pure state and is detrimental to the state discrimination for lower dimensional systems; while it is the opposite for the mixed-mixed case with symmetrically distributed coherence. Making an extension to infinite-dimensional systems, we find that the coherence which is detrimental to state discrimination may become helpful and vice versa.

## 1. Introduction

Quantum state discrimination is of great importance in quantum information processing [1]. A fundamental result in quantum mechanics is the impossibility to distinguish perfectly two or more non-orthogonal quantum states. It is then a key task to discriminate the states with maximal success probability. Such state discrimination problems branch out into two important streams: ambiguous [2,3,4,5,6] and unambiguous quantum state discrimination [7,8,9,10,11,12,13,14,15,16,17]. The study on minimization of the error in the ambiguous state discrimination was pioneered by Helstrom who provided a lower bound on the error probability in distinguishing two quantum states. This bound can be attained through the ways presented in [2,3,4]. While the unambiguous quantum state discrimination is error-free [7,8,9,10,11,12]. It plays key roles in various contexts in quantum information theory, including quantum key distribution [11,13,16], the study of quantum correlations [18,19,20,21], and the role of entanglement in local discrimination of bipartite systems [22].

Quantum coherence is also a critical resource in quantum state discrimination and is tightly related to quantum correlations such as quantum entanglement [23]. Recently, the quantification of quantum coherence has been extensively studied in the framework of quantum resource theory [24,25,26,27]. The role of coherence played in ambiguous state discriminations [5,6] has been investigated. There are also a few results on unambiguous state discriminations with coherence which is generated or consumed in auxiliary systems and utilized as resources [28]. Actually, the coherence in [28] comes from the non-orthogonality of the initial states.

In this work, different from the results in [28], we consider the effect of the coherence encoded in the initial state on unambiguous state discriminations. We first apply a quantum state filtering [29], which is the discrimination between a pure state from another rank-*N* incoherent mixed state composed of *N* vectors. Then, we superpose these *N* vectors into a new pure state and then do a pure-pure state discrimination. If the fidelity of the pure-pure state equals the pure-mixed one, it can be proved that the pure-pure scheme is superior to the pure-mixed one; but the coherence is detrimental to the state discrimination for lower dimensional systems. Furthermore, through the discrimination of two rank-*N* mixed states and the comparison with the results of another pure-pure-state discrimination scheme, as an extension of the results in [22], we prove that pure-pure scheme is still superior to mixed-mixed one if the eigenvectors of the mixed states have a one-to-one overlap (an equal-fidelity case); but there exists a great deal of symmetrically distributed coherence which is helpful to state discrimination, in contrary to the result of quantum filtering.

Finally, we extend the results to infinite-dimensional systems where the vectors included in the mixed states are mixed with each other via the probability factors coinciding with the photon number distribution of two kinds of Gaussian states in quantum optics [30]. We find that corresponding to the well-known coherent state, the symmetrically (asymmetrically) distributed coherence may become detrimental (helpful) on the contrary, which can be attributed to the fact that the well-known coherent state approaches the boundary between classical and quantum physics.

The paper is organized as follows. In Section 2, we present the result of quantum state filtering. Additionally, we compare its results with the one of the pure-pure state schemes. In Section 3, we compare the discrimination of two rank-*N* mixed states with the scheme for discriminating other two pure states having the same fidelity with the mixed ones. We generalize the results to infinite systems associated with two kinds of Gaussian states in Section 4. We summarize in the last section.

## 2. Quantum State Filtering

Consider a set of given N+1 non-orthogonal quantum states $\{|\Psi_{1}\rangle ,|\Psi_{i^{\prime }}\rangle \}$ (i=1,2,...,N), occurring with prior probability $P_{1},P_{2}\beta_{i}$, where $P_{1}+P_{2}=1$ and $\sum_{i=1}^{N}\beta_{i}=1$, $\beta_{i}\geq 0$. We want to find a procedure that unambiguously assigns the state of the quantum system to one or the other of two complementary subsets of the set of the N+1 given non-orthogonal quantum states, namely, either $|\Psi_{1}\rangle$ or $\left\{|\Psi_{i^{\prime }}\rangle \right\}$. This is called quantum filtering (pure-mixed state discrimination) [29] which is equivalent to the problem of discrimination between a pure (rank-1) state $\rho_{1}$ and an rank-*N* mixed state $\rho_{2}$,

(1) $$\begin{array}{ccc} \rho_{1} & = & |\Psi_{1}\rangle \langle \Psi_{1}|,\\ \rho_{2} & = & \sum_{i=1}^{N}\beta_{i}|\Psi_{i^{\prime }}\rangle \langle \Psi_{i^{\prime }}|, \end{array}$$

prepared with the prior probability $P_{1}$ and $P_{2}$ ($P_{1}\leq P_{2}$). For simplicity, we assume that the following relations are fulfilled:

(2) $$\langle \Psi_{1}|\Psi_{i^{\prime }}\rangle =s_{1i^{\prime }}\geq 0,\;\;\langle \Psi_{i^{\prime }}|\Psi_{j^{\prime }}\rangle =\delta_{ij}$$

for i,j=1,...,N. Let $|\Psi_{1}^{\|}\rangle$ be the component of $|\Psi_{1}\rangle$ in the subspace spanned by the vectors $|\Psi_{1^{\prime }}\rangle ,|\Psi_{2^{\prime }}\rangle ,...,|\Psi_{n^{\prime }}\rangle$. We have

(3) $$\langle \Psi_{1}^{\|}|\Psi_{1}^{\|}\rangle =\sum_{i=1}^{N}\overset{2}{\underset{1i^{\prime }}{s}}<1.$$

In order to discriminate the two sets unambiguously, we couple the system with an ancilla $|k_{a}\rangle$ [15,17,29] via the tensor product method [31] and perform a joint unitary transformation *U*,

(4) $$\begin{array}{ccc} U|\Psi_{1}\rangle |k_{a}\rangle \;\; & = & \;\;\sqrt{q_{1}}e^{i\gamma_{1}}|\phi_{0}{\rangle |0\rangle }_{a}+\sqrt{1-q_{1}}|\Phi_{1}{\rangle |1\rangle }_{a},\\ U|\Psi_{i^{\prime }}\rangle |k_{a}\rangle \;\; & = & \;\;\sqrt{q_{i^{\prime }}}e^{i\gamma_{i^{\prime }}}|\phi_{0}{\rangle |0\rangle }_{a}+\sqrt{1-q_{i^{\prime }}}|\Phi_{i^{\prime }}{\rangle |1\rangle }_{a}. \end{array}$$

Since we are aiming to discriminate $|\Psi_{1}\rangle$ from $|\Psi_{i^{\prime }}\rangle$ optimally, it is required that the post-measured state $|\Phi_{1}\rangle$ is orthogonal to $|\Phi_{i^{\prime }}\rangle$, $\langle \Phi_{1}|\Phi_{i^{\prime }}\rangle =0$ for i=1,2,...,N, while $\langle \Phi_{i^{\prime }}|\Phi_{j^{\prime }}\rangle \neq 0$ for i≠j, i,j=1,2,...,N. Thus, after a von-Neumann measurement on the ancilla, the vector $|\Psi_{1}\rangle$ is distinguished from the set $\left\{|\Psi_{i^{\prime }}\rangle \right\}$ successfully if the measurement outcome is ${|1\rangle }_{a}$, otherwise the outcome ${|0\rangle }_{a}$ implies failure. The average failure probability *Q* is given by

(5) $$Q=P_{1}q_{1}+\sum_{i=1}^{N}P_{2}\beta_{i}q_{i^{\prime }},$$

where the parameters $q_{1}$ and $q_{i^{\prime }}$ satisfy $q_{1}q_{i^{\prime }}=\overset{2}{\underset{1i^{\prime }}{s}}$ according to Equation (4). Therefore, the optimization of *Q* is given by

(6) $$\mathrm{minimize}\;Q=P_{1}q_{1}+\frac{\sum_{i=1}^{N}P_{2}\beta_{i}s_{1i^{\prime }}^{2}}{q_{1}},$$

(7) $$\mathrm{subject}\;\mathrm{to}\;\;q_{1}\in \left[\langle \Psi_{1}^{\|}|\Psi_{1}^{\|}\rangle ,\;1\right],$$

where $|\Psi_{1}^{\|}\rangle$ is the component of $|\Psi_{1}\rangle$ which lies in the subspaces spanned by $\{|\Psi_{1^{\prime }}\rangle$...$|\Psi_{N^{\prime }}\rangle \}$. The constraint (7) for the quantum filtering is acquired based on the semidefinite property of the Gram matrix given by the vectors $\{|\Phi_{1}\rangle ,|\Phi_{1^{\prime }}\rangle ,...,|\Phi_{i^{\prime }}\rangle \}$ [29]. Set $S=(s_{11^{\prime }},s_{12^{\prime }},...,s_{1N^{\prime }})$. We have the optimal solution,

(8a) $$\begin{array}{ccc} (i):\; & Q_{\min}\;\;=\;\;2\sqrt{P_{1}P_{2}\;\;\sum_{i=1}^{N}\beta_{i}s_{1i^{\prime }}^{2}\;},\;\; & \mathrm{when}\;S\in \Lambda_{\left(i\right)}; \end{array}$$

(8b) $$\begin{array}{ccc} (\mathrm{ii}):\; & Q_{\min}\;\;=\;\;P_{1}\;\langle \Psi_{1}^{\|}\;|\;\Psi_{1}^{\|}\rangle \;\;+\;\;\frac{P_{2}\;\left(\;\sum_{i=1}^{N}\beta_{i}s_{1i^{\prime }}^{2}\right)\;\;}{\;\langle \Psi_{1}^{\|}|\Psi_{1}^{\|}\rangle \;},\;\; & \mathrm{when}\;S\in \Lambda_{\left(ii\right)}; \end{array}$$

(8c) $$\begin{array}{ccc} (\mathrm{iii}):\; & Q_{\min}\;=\;P_{1}\;+\;P_{2}\left(\sum_{i=1}^{N}\beta_{i}\overset{2}{\underset{1i^{\prime }}{s}}\right),\;\; & \mathrm{when}\;S\in \Lambda_{\left(iii\right)}, \end{array}$$

where

(9a) $$\begin{array}{cc} \; & \Lambda_{\left(i\right)}=\left\{S:\;\langle \Psi_{1}^{\|}|\Psi_{1}^{\|}\rangle \leq q_{1}^{*}\leq 1\right\}, \end{array}$$

(9b) $$\begin{array}{cc} \; & \Lambda_{\left(ii\right)}=\left\{S:\;0<q_{1}^{*}<\langle \Psi_{1}^{\|}|\Psi_{1}^{\|}\rangle \right\}, \end{array}$$

(9c) $$\begin{array}{cc} \; & \Lambda_{\left(iii\right)}=\left\{S:\;q_{1}^{*}>1\right\}, \end{array}$$

and

(10) $$q_{1}^{*}=\sqrt{\frac{P_{2}}{P_{1}}\sum_{i=1}^{N}\beta_{i}s_{1i^{\prime }}^{2}}.$$

Corresponding to this optimal solution, for case (i) and (ii), both $|\Psi_{1}\rangle$ and $|\Psi_{i^{\prime }}\rangle$ (i=1,2,...,N) are identified; for case (iii), the state $|\Psi_{1}\rangle$ is required to be neglected.

Since the fidelity has been found to be closely correlated with the state discrimination problems [32], Terry et al. [33] give a lower bound of the optimal failure probability for the quantum filtering scheme,

(11) $$Q_{\min}=2\sqrt{P_{1}P_{2}}F\left(\rho_{1},\rho_{2}\right),$$

where $F(\rho_{1},\rho_{2})$ is the fidelity between $\rho_{1}$ and $\rho_{2}$ given by [33],

(12) $$\begin{array}{ccc} F(\rho_{1},\rho_{2}) & = & F(|\Psi_{1}\rangle \langle \Psi_{1}|,\rho_{2})\\ & = & \sqrt{\langle \Psi_{1}|\rho_{2}|\Psi_{1}\rangle }=\sqrt{\sum_{i=1}^{N}\beta_{i}s_{1i^{\prime }}^{2}}. \end{array}$$

One can see that this lower bound is saturated for case (i) in Equation (8a).

To see the essential difference between quantum superposition and classical mixture, and the role played by quantum coherence in high-dimensional mixed state discrimination, we replace the classical probability $\beta_{i}$ with quantum probability amplitudes. Then, the vectors $|\Psi_{i}^{\prime }\rangle$ (i=1,...,N) in the decomposition of $\rho_{2}$ are superposed into a pure state [22],

(13) $$|\Psi_{2}\rangle =\sum_{i=1}^{N}\sqrt{\beta_{i}}e^{i\theta_{i}}|\Psi_{i^{\prime }}\rangle ,$$

where $0\leq \theta_{i}\leq 2\pi$.

After the first results on characterization and quantification of coherence [24,25], Baumgratz et al. [34] put forward the resource-theoretic framework of coherence and formulated a set of axioms or preconditions for a measure of coherence. As the bona fide measures for coherence, the $l_{1}$ norm of coherence is defined by

(14) $$C_{l_{1}}\left(\rho \right)=\sum_{i\neq j}\left|\rho_{ij}\right|,$$

and the relative entropy of coherence is given by

(15) $$C_{\mathrm{rel}}\left(\rho \right)=S\left(\rho_{\mathrm{diag}}\right)-S\left(\rho \right),$$

where $\rho =\sum_{ij}\rho_{ij}\left|i\rangle \langle j\right|$ is the density matrix and $\rho_{\mathrm{diag}}=\sum_{ii}\rho_{ii}\left|i\rangle \langle i\right|$ is the diagonal part of ρ. Both $l_{1}$ norm and relative entropy coherence measures are bases dependent. Below we consider the coherence under the fixed orthogonal basis $\left\{|\Psi_{i^{\prime }}\rangle \right\}$ given in $\rho_{2}$. Thus, the $l_{1}$ norm coherence of $|\Psi_{2}\rangle$ is given by

(16) $$C_{l_{1}}(|\Psi_{2}\rangle )=\sum_{i\neq j}\left|\rho_{ij}\right|=2\sum_{i>j}^{N}\sqrt{\beta_{i}\beta_{j}}.$$

The failure probability corresponding to the optimal discrimination between $|\Psi_{1}\rangle$ and $|\Psi_{2}\rangle$ is

(17a) $$\begin{array}{ccc} (i^{\prime }):\; & Q_{\min}^{\prime }=2\sqrt{P_{1}P_{2}}\left|s^{*}\right|,\;\; & \;\;\;\mathrm{when}\;S\in \Lambda_{\left(i^{\prime }\right)}, \end{array}$$

(17b) $$\begin{array}{ccc} ({\mathrm{ii}}^{\prime }):\; & Q_{\min}^{\prime }=P_{1}+P_{2}{\left|s^{*}\right|}^{2},\;\; & \;\;\;\mathrm{when}\;S\in \Lambda_{\left(ii^{\prime }\right)}, \end{array}$$

where

(18) $$\begin{array}{ccc} s^{*} & = & \langle \Psi_{1}|\Psi_{2}\rangle =\sum_{i=1}^{N}\sqrt{\beta_{i}}s_{1i^{\prime }}e^{i\theta_{i}},\\ \Lambda_{\left(i^{\prime }\right)} & = & \{S:0\leq |s^{*}|\leq \sqrt{\frac{P_{1}}{P_{2}}}\},\\ \Lambda_{\left(ii^{\prime }\right)} & = & \{S:\sqrt{\frac{P_{1}}{P_{2}}}<|s^{*}|\leq 1\}. \end{array}$$

**Figure 1:**
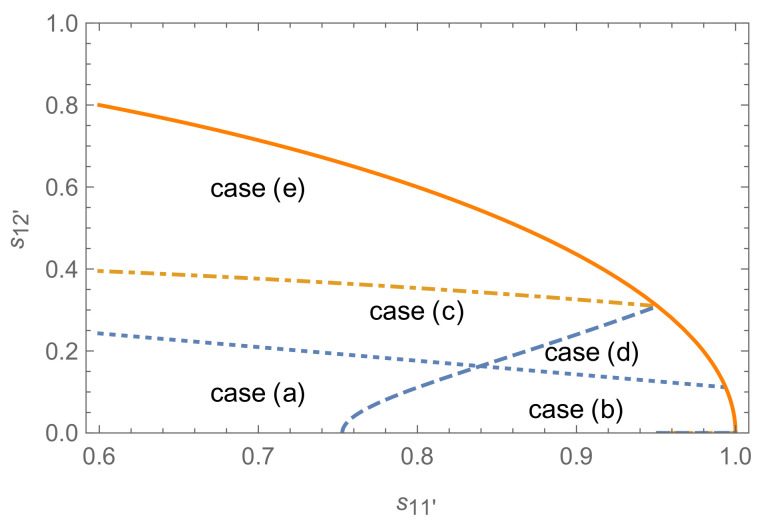
Set $P_{1}=0.15$ and $\beta_{1}=0.1$. For N=2, we have five regions corresponding to cases (a)–(e), respectively, with respect to different values of $s_{11^{\prime }}$ and $s_{12^{\prime }}$. The dashed line for $q_{1}^{*}=s_{11^{\prime }}^{2}+s_{12^{\prime }}^{2}$, dot-dashed line is for $q_{1}^{*}=1$, dotted line for $s^{*}=\sqrt{\frac{P_{1}}{P_{2}}}$, and orange solid line for $s_{11^{\prime }}^{2}+s_{12^{\prime }}^{2}=1$.

Focusing on the difference between the result of classical mixture and quantum superposition, we consider $\Delta Q=Q_{\min}-\overset{\prime }{\underset{\min}{Q}}$ with respect to the following five cases, as the case $S\in \Lambda_{\left(i^{\prime }\right)}\cap \Lambda_{\left(iii\right)}$ corresponds to an empty set according to (9) and (18),

(19) $$\begin{array}{ccc} \mathrm{case}\;(a):\;S & \in & \Lambda_{\left(i^{\prime }\right)}\cap \Lambda_{\left(i\right)};\;\mathrm{case}\;(b):\;S\in \Lambda_{\left(i^{\prime }\right)}\cap \Lambda_{\left(ii\right)};\\ \mathrm{case}\;(c):\;S & \in & \Lambda_{\left(ii^{\prime }\right)}\cap \Lambda_{\left(i\right)};\;\mathrm{case}\;(d):\;S\in \Lambda_{\left(ii^{\prime }\right)}\cap \Lambda_{\left(ii\right)};\\ \mathrm{case}\;(e):\;S & \in & \Lambda_{\left(ii^{\prime }\right)}\cap \Lambda_{\left(iii\right)}, \end{array}$$

see Figure 1 for N=2, $P_{1}=0.15$ and $\beta_{1}=0.1$.

To find out the role played by quantum supposition in our state discrimination, we consider the difference of the optimal success probability between the pure-pure and pure-mixed state discrimination. We have the following theorem.

**Theorem** **1.** *The minimum failure probability $Q_{\min}^{\prime }$ of pure-pure state discrimination is upper bounded by the one of quantum state filtering $Q_{\min}$, namely, $\Delta Q=Q_{\min}-Q_{\min}^{\prime }\geq 0$, if the following equal-fidelity condition holds, $F\left(\rho_{1},\rho_{2}\right)=F(|\Psi_{1}\rangle ,|\Psi_{2}\rangle )$.*

**Proof**  (Proof of Theorem 1). Since the fidelity between the two pure states $|\Psi_{1}\rangle$ and $|\Psi_{2}\rangle$ is given by

(20) $$F(|\Psi_{1}\rangle ,|\Psi_{2}\rangle )=|s^{*}|,$$

combining with Equations (8), (10), (12), (17) and (18), we have the following results corresponding to the five different cases listed in (19). With respect to the case (a), we have

(21) $$\begin{array}{ccc} {\left(\Delta Q\right)}^{2} & = & Q_{\min}^{2}-Q_{\min}^{\prime 2}\\ & = & 4P_{1}P_{2}\sum_{i=1}^{N}\beta_{i}s_{1i^{\prime }}^{2}-4P_{1}P_{2}{\left|s^{*}\right|}^{2}\\ & = & 4P_{1}P_{2}[F^{2}\left(\rho_{1},\rho_{2}\right)\;-\;F^{2}(|\Psi_{1}\rangle ,|\Psi_{2}\rangle )]\;=\;0. \end{array}$$

For the case (b), we have

(22) $$\begin{array}{ccc} \Delta Q & = & P_{1}\langle \Psi_{1}^{\|}|\Psi_{1}^{\|}\rangle +P_{2}\frac{\sum_{i=1}^{N}\beta_{i}s_{1i^{\prime }}^{2}}{\langle \Psi_{1}^{\|}|\Psi_{1}^{\|}\rangle }\;-\;2\sqrt{P_{1}P_{2}}\left|s^{*}\right|\\ & = & P_{1}\;\langle \Psi_{1}^{\|}|\Psi_{1}^{\|}\;\rangle \;+\;P_{2}\frac{F^{2}\left(\rho_{1},\rho_{2}\right)}{\langle \Psi_{1}^{\|}|\Psi_{1}^{\|}\rangle }\;-\;2\;\sqrt{P_{1}P_{2}}\;F(\;|\Psi_{1}\rangle \;,\;|\Psi_{2}\rangle \;)\\ & = & {\left[\sqrt{P_{1}\langle \overset{\|}{\underset{1}{\Psi }}|\overset{\|}{\underset{1}{\Psi }}\rangle }-\sqrt{\frac{P_{2}}{\langle \overset{\|}{\underset{1}{\Psi }}|\overset{\|}{\underset{1}{\Psi }}\rangle }}F\left(\rho_{1},\rho_{2}\right)\right]}^{2}\geq 0. \end{array}$$

Corresponding to the case (c), we obtain that

(23) $$\begin{array}{ccc} S & \in & \Lambda_{\left(ii^{\prime }\right)}\cap \Lambda_{\left(i\right)}\\ & = & \left\{S:\right|s^{*}\left|>\sqrt{\frac{P_{1}}{P_{2}}}\right\}\cap \left\{S:\langle \Psi_{1}^{\|}|\Psi_{1}^{\|}\rangle \leq q_{1}^{*}\leq 1\right\}\\ & = & \{S:F(|\Psi_{1}\rangle ,|\Psi_{2}\rangle )>\sqrt{\frac{P_{1}}{P_{2}}}\}\cap \left\{S:\sqrt{\frac{P_{1}}{P_{2}}}\langle \;\Psi_{1}^{\|}|\Psi_{1}^{\|}\rangle \;\leq \;F\left(\rho_{1},\rho_{2}\right)\;\leq \;\sqrt{\frac{P_{1}}{P_{2}}}\right\}, \end{array}$$

which is just an empty set under the equal-fidelity condition. For the case (d) we have

(24) $$\begin{array}{ccc} S & \in & \Lambda_{\left(ii^{\prime }\right)}\cap \Lambda_{\left(ii\right)}\\ & = & \{S:\sqrt{\frac{P_{1}}{P_{2}}}<|s^{*}\left|\leq 1\right\}\cap \left\{S:0<q_{1}^{*}<\langle \Psi_{1}^{\|}|\Psi_{1}^{\|}\rangle \right\}\\ & = & \{S:F(|\Psi_{1}\rangle ,|\Psi_{2}\rangle )>\sqrt{\frac{P_{1}}{P_{2}}}\}\cap \left\{S:0<F\left(\rho_{1},\rho_{2}\right)<\sqrt{\frac{P_{1}}{P_{2}}}\langle \Psi_{1}^{\|}|\Psi_{1}^{\|}\rangle \right\}, \end{array}$$

which is again an empty set under the equal-fidelity condition. With respect to the case (e), we get

(25) $$\begin{array}{ccc} \Delta Q & = & P_{1}+P_{2}\sum_{i=1}^{N}\beta_{i}s_{1i^{\prime }}^{2}-(P_{1}+P_{2}|s^{*}{|}^{2})\\ & = & P_{2}[F^{2}\left(\rho_{1},\rho_{2}\right)-F^{2}(|\Psi_{1}\rangle ,|\Psi_{2}\rangle )]=0. \end{array}$$

From the above results, we have that $\Delta Q=Q_{\min}-Q_{\min}^{\prime }\geq 0$ under the equal-fidelity condition $F\left(\rho_{1},\rho_{2}\right)=F(|\Psi_{1}\rangle ,|\Psi_{2}\rangle )$. □

From the proof of Theorem 1, we see that the superiority of a pure-pure state scheme versus a pure-mixed one may only possibly occur for case (b). Concerning the equal-fidelity condition in Theorem 1, we have the following conclusion.

**Corollary** **1.** *For the comparison of pure-mixed and pure-pure state discrimination scheme, the equal-fidelity condition $F(|\Psi_{1}\rangle ,|\Psi_{2}\rangle )=F\left(\rho_{1},\rho_{2}\right)$ is satisfied if and only if*

(26) $$\sum_{i>j}^{N}\sqrt{\beta_{i}\beta_{j}}s_{1i^{\prime }}s_{1j^{\prime }}\cos\left(\theta_{i}-\theta_{j}\right)=0.$$

As for an illustration, consider N=2, $s_{1i^{\prime }}\neq 0$ and $s_{1j^{\prime }}\neq 0$. According to Equation (26), we have

(27) $$\cos(\theta_{1}-\theta_{2})=0.$$

Then, from Equations (9), (19) and (27), the case (b) is also rejected. Namely, ΔQ>0 is impossible in this situation. As for another example, let us consider the following case,

(28) $$s_{1i^{\prime }}=\delta_{it}s_{1t},$$

which satisfies the equal-fidelity relation (26) obviously. We have

$$\Delta Q={\left(\sqrt{P_{1}}s_{1t^{\prime }}-\sqrt{P_{2}\beta_{t}}\right)}^{2}.$$

**Figure 2:**
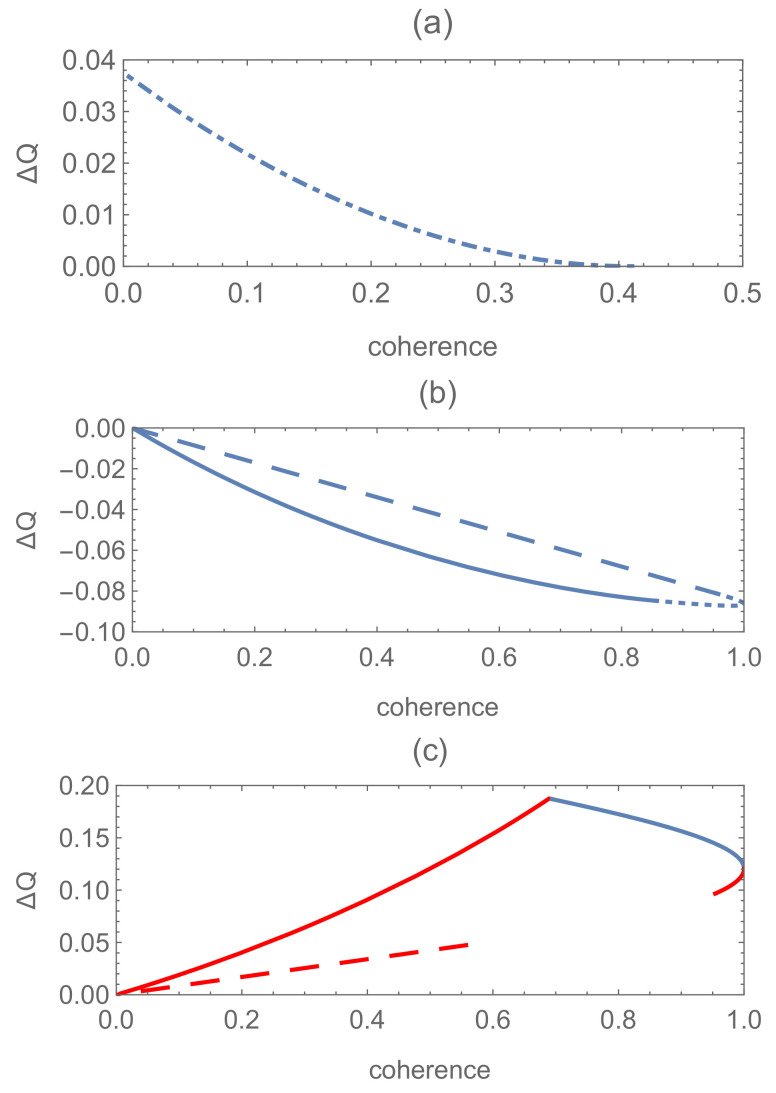
The difference ΔQ of the optimal success probabilities between the pure-mixed and pure-pure schemes as functions of the coherence encoded in the state $|\Psi_{2}\rangle$ for N=2, $P_{1}=0.15$ and $s_{12^{\prime }}=0.5$. (**a**–**c**) correspond to equal-fidelity case ($s_{11^{\prime }}=0$), equal-phase case ($s_{11^{\prime }}=0.2$, $\theta_{1}=\theta_{2}=0$) and unequal-phase case ($s_{11^{\prime }}=0.2$, $\theta_{1}=\pi /2$, $\theta_{2}=-\pi /2$), respectively. Solid, dotted, dashed, and dot-dashed lines correspond to the cases (a), (b), (d) and (e) in Figure 1, respectively; while the case (**c**) does not match with here. Blue (red) lines correspond to the coherence detrimental (helpful) to state discrimination (the same for Figure 3, Figure 4, Figure 5, Figure 6 and Figure 7).

Figure 2a shows the relations between ΔQ and the coherence in this case.

Instead of the equal-fidelity condition, if we set the all phases in Equation (13) to equal to each other, $\theta_{i}=\theta_{j}$ (i≠j, and i,j=1,2,...,N), then we have the following theorem.

**Theorem** **2.** *If $\theta_{i}=\theta_{j}$, the pure-pure state discrimination scheme is inferior to quantum state filtering, i.e., $\Delta Q=Q_{\min}-Q_{\min}^{\prime }\leq 0$ for all the cases except for the case (b). When $s_{1i^{\prime }}=s_{1j^{\prime }}=s_{0}$, ΔQ ($\Delta Q^{2}$) is proportional to $C_{l_{1}}(|\Psi_{1}\rangle )$ for the case (e) (case (a)), and the upper bound of ΔQ is proportional to $C_{l_{1}}(|\Psi_{1}\rangle )$ for cases (c) and (d).*

**Proof**  (Proof of Theorem 2). For cases (a), (b) and (d), the expressions of ΔQ are the same as the ones in Equations (21), (22) and (25). For the case (a), we have

$$\begin{array}{ccc} \Delta Q^{2} & = & Q_{\min}^{2}-Q_{\min}^{\prime 2}=-4P_{1}P_{2}\sum_{i\neq j}\sqrt{\beta_{i}\beta_{j}}s_{1i^{\prime }}s_{1j^{\prime }}e^{i(\theta_{i}-\theta_{j})}\\ & = & -8P_{1}P_{2}\sum_{i>j}\sqrt{\beta_{i}\beta_{j}}s_{1i^{\prime }}s_{1j^{\prime }}. \end{array}$$

For $s_{1i^{\prime }}=s_{1j^{\prime }}=s_{0}$, we have

(29) $$\Delta Q^{2}=-8P_{1}P_{2}s_{0}^{2}\sum_{i>j}^{N}\sqrt{\beta_{i}\beta_{j}}=-4P_{1}P_{2}s_{0}^{2}C_{l_{1}}(|\Psi_{2}\rangle )<0.$$

Similarly, for the case (c), we have

$$\Delta Q=P_{1}\langle \Psi_{1}^{\|}|\Psi_{1}^{\|}\rangle +P_{2}\frac{\sum_{i=1}^{N}\beta_{i}s_{1i^{\prime }}^{2}}{\langle \Psi_{1}^{\|}|\Psi_{1}^{\|}\rangle }-(P_{1}\;+\;P_{2}|s^{*}{|}^{2}).$$

According to that $\langle \Psi_{1}^{\|}|\Psi_{1}^{\|}\rangle \leq 1$, we have

$$\begin{array}{ccc} \Delta Q & \leq & P_{1}+P_{2}\sum_{i=1}^{N}\beta_{i}s_{1i^{\prime }}^{2}-(P_{1}+P_{2}|\sum_{i=1}^{N}\sqrt{\beta_{i}}s_{1i^{\prime }}e^{i\theta_{i}}{|}^{2})\\ & = & -2P_{2}\sum_{i>j}^{N}\sqrt{\beta_{i}\beta_{j}}s_{1i^{\prime }}s_{1j^{\prime }}\cos\left(\theta_{i}-\theta_{j}\right)=-P_{2}\overset{2}{\underset{0}{s}}C_{l_{1}}(|\Psi_{2}\rangle )<0. \end{array}$$

For the case (d), we get

$$\begin{array}{ccc} \Delta Q & = & 2\sqrt{P_{1}P_{2}\sum_{i=1}^{N}\beta_{i}\overset{2}{\underset{1i^{\prime }}{s}}}-P_{1}-P_{2}{\left|\sum_{i=1}^{N}\sqrt{\beta_{i}}s_{1i^{\prime }}e^{i\theta_{i}}\right|}^{2}\\ & \leq & P_{1}+P_{2}\sum_{i=1}^{N}\beta_{i}\overset{2}{\underset{1i^{\prime }}{s}}-P_{1}-P_{2}{\left|\sum_{i=1}^{N}\sqrt{\beta_{i}}s_{1i^{\prime }}e^{i\theta_{i}}\right|}^{2}\\ & = & -2P_{2}\sum_{i>j}^{N}\sqrt{\beta_{i}\beta_{j}}s_{1i^{\prime }}s_{1j^{\prime }}\cos\left(\theta_{i}-\theta_{j}\right)=-P_{2}\overset{2}{\underset{0}{s}}C_{l_{1}}(|\Psi_{2}\rangle )<0. \end{array}$$

For the case (e), we obtain

$$\begin{array}{ccc} \Delta Q & = & P_{1}+P_{2}\sum_{i=1}^{N}\beta_{i}\overset{2}{\underset{1i^{\prime }}{s}}-[P_{1}+P_{2}|\sum_{i=1}^{N}\sqrt{\beta_{i}}s_{1i^{\prime }}e^{i\theta_{i}}{|}^{2}]\\ & = & -2P_{2}\sum_{i>j}^{N}\sqrt{\beta_{i}\beta_{j}}s_{1i^{\prime }}s_{1j^{\prime }}\cos\left(\theta_{i}-\theta_{j}\right)=-P_{2}\overset{2}{\underset{0}{s}}C_{l_{1}}(|\Psi_{2}\rangle )<0. \end{array}$$

□

The inequality ΔQ<0 does not always hold under the condition in Theorem 2 for the case (b), which also can be seen from Theorem 1, where it is indicated that ΔQ≥0 under the equal-fidelity condition. To illustrate the role played by the quantum coherence in our procedure, we show the difference ΔQ as a function of coherence of $|\Psi_{2}\rangle$ in Figure 2. One can see that for the equal-phase cases shown in Figure 2a,b, the quantum coherence is not a critical recourse but detrimental to the unambiguous state discrimination even for the cases where the pure-pure state scheme is superior to pure-mixed one as guaranteed by the equal-fidelity condition. This result is different from the one in [28] where the coherence generated in the auxiliary system is positively correlated with the optimal success probability of state discrimination.

Nevertheless, when the phases $\theta_{i}$ turn to be unequal (shown in Figure 2c), the following two conclusions may be drawn: (i) the optimal success probability of the pure-mixed scheme may be surpassed by the pure-pure state one, on the contrary; (ii) some of the coherence encoded in the pure state is not detrimental but helpful to state discrimination (shown in Figure 2c). Namely, one can acquire helpful coherence via adjusting the phase factors in the superposed state $|\Psi_{2}\rangle$. By a straightforward calculation, it is easily known that this superiority of the pure-pure state discrimination scheme versus the pure-mixed one, as shown in Figure 2c, can be attributed to the fact that $F\left(\rho_{1},\rho_{2}\right)\geq F(|\Psi_{1}\rangle ,|\Psi_{2}\rangle )$ according to Equations (12) and (20). The reverse is also true for the results in Figure 2b. Since the state with a lower fidelity is easier to be discriminated, the superiority of pure-pure state discrimination versus pure-mixed one occurs without surprise. Then, we try to find some significant results by comparison of mixed-mixed versus pure-pure state discrimination under equal-fidelity conditions in the following section.

## 3. Discrimination of Two Rank-*N* Mixed States

We have studied the quantum filtering problem as a special instance for the discrimination of two mixed states. It indicates that a pure-pure state discrimination scheme with a same fidelity as the pure-mixed one tends to be more possible to succeed. This prompts us to investigate the discrimination of two rank-*N* mixed states of the following form,

(30) $$\rho_{1}=\sum_{i=1}^{N}\alpha_{i}|\Psi_{i}\rangle \langle \Psi_{i}|,\;\;\;\rho_{2}=\sum_{i=1}^{N}\beta_{i}|\Psi_{i^{\prime }}\rangle \langle \Psi_{i^{\prime }}|,$$

where $\alpha_{i}>0$, $\beta_{i}>0$, $\sum_{i=1}^{N}\alpha_{i}=\sum_{i=1}^{N}\beta_{i}=1$. The orthonormal bases $\left\{|\Psi_{i}\rangle \right\}$ and $\left\{|\Psi_{i^{\prime }}\rangle \right\}$ satisfy

(31) $$\langle \Psi_{i}|\Psi_{j}\rangle \;=\;\delta_{ij},\;\langle \Psi_{i^{\prime }}|\Psi_{j^{\prime }}\rangle \;=\;\delta_{ij},\;\langle \Psi_{i}|\Psi_{j^{\prime }}\rangle \;=\;s_{ii^{\prime }}\delta_{ij}$$

with i,j=1,...,N and $s_{ii^{\prime }}=\langle \Psi_{i}|\Psi_{i^{\prime }}\rangle >0$. The state $\rho_{i}$ occurs with *a priori* probability $P_{i}$ (i=1,2, $P_{1}+P_{2}=1$, $P_{1}\alpha_{i}\leq P_{2}\beta_{i}$).

The relation (31) means that the vectors composing $\rho_{1}$ are one to one overlapped with the ones of $\rho_{2}$ and is satisfied for the following example:

(32) $$|\Psi_{i}\rangle \;=\;|2i\;-\;2\rangle ,\;\;\;|\Psi_{i^{\prime }}\rangle \;=\;s_{ii^{\prime }}|2i\;-\;2\rangle +\sqrt{1\;-\;|s_{ii^{\prime }}{|}^{2}}|2i\;-\;1\rangle ,$$

where {|2i−1〉} and {|2i〉} (i=1,...,N) are orthonormal bases in a 2N dimensional Hilbert space.

If we compare the above results with the discrimination of a pair of the following pure states,

(33) $$|\Phi_{1}\rangle =\sum_{i=1}^{N}\sqrt{\alpha_{i}}|\Psi_{i}\rangle ,\;\;\;|\Phi_{2}\rangle =\sum_{i=1}^{N}\sqrt{\beta_{i}}|\Psi_{i^{\prime }}\rangle ,$$

occurring with a priori probability $P_{1}$ and $P_{2}$, respectively, the relation (31) guarantees the equal-fidelity condition

$$F\left(\rho_{1},\rho_{2}\right)=F(|\Phi_{1}\rangle ,|\Phi_{2}\rangle )=\sum_{i=1}^{N}\sqrt{\alpha_{i}\beta_{i}}\langle \Psi_{i}|\Psi_{i^{\prime }}\rangle .$$

The conditions in Equation (31) also ensure that the discrimination of $\rho_{1}$ and $\rho_{2}$ can be carried out in *N* independent subspaces through optimal POVM operators which can be written as a direct sum of *N* corresponding parts, just like the results for the discrimination of rank-two mixed state in [14,22]. Then, concerning the optimal discrimination of $\rho_{1}$ and $\rho_{2}$, we have the following remark.

**Remark** **1.** *The successful probability of discrimination between the two rank-N states $\rho_{1}$ and $\rho_{2}$ in Equation (30) satisfying the relation (31) is equivalent to a weighed average of the one for the discrimination between the ith pair of eigenvectors $\{|\Psi_{i}\rangle ,|\Psi_{i^{\prime }}\rangle \}$ (i=1,...,N).*

Thus, if $0<s_{ii^{\prime }}\leq \sqrt{\frac{P_{1}\alpha_{i}}{P_{2}\beta_{i}}}$ (1≤i≤m), $\sqrt{\frac{P_{1}\alpha_{i}}{P_{2}\beta_{i}}}<s_{ii^{\prime }}\leq 1$ (m+1≤i≤N), where *m* is an integer satisfies 1<m<N, the minimum failure probability for discriminating $\rho_{1}$ from $\rho_{2}$ is given by

$$Q_{\min}=\sum_{i=1}^{m}2\sqrt{P_{1}P_{2}\alpha_{i}\beta_{i}}s_{ii^{\prime }}\;+\;\sum_{i=m+1}^{N}\left(P_{1}\alpha_{i}\;+\;P_{2}\beta_{i}s_{ii^{\prime }}^{2}\right).$$

Here, the vectors {$|\Psi_{i}\rangle ,|\Psi_{i^{\prime }}\rangle$} (1≤i≤m) are all identified while $|\Psi_{i}\rangle$ (m<i≤N) are neglected in the optimal solution for the discrimination between $\rho_{1}$ and $\rho_{2}$.

For the discrimination of pure-pure states, optimal failure probability is of the same form as Equation (17). Set

(34) $$s^{*}=\sum_{i=1}^{N}\sqrt{\alpha_{i}\beta_{i}}s_{ii^{\prime }}.$$

We have the following theorem as an extension of the work for the discriminating rank-two mixed states in [22].

**Theorem** **3.** *For the discrimination of two rank-N mixed states in Equation (30), the minimum failure probability $Q_{\min}^{\prime }$ corresponding to the optimal discrimination of the pure states in Equation (33) is upper bounded by $Q_{\min}$ for the mixed states.*

**Proof** **of** **Theorem** **3.** Corresponding to different values of $s_{ii^{\prime }}$, we have the following four cases.

Case (i): $0<s_{ii^{\prime }}\leq \sqrt{\frac{P_{1}\alpha_{i}}{P_{2}\beta_{i}}}$, which implies that $s^{*}\leq \sqrt{\frac{P_{1}}{P_{2}}}$ (i=1,2,...,N). Here, all vectors included in $\rho_{1}$ and $\rho_{2}$ are identified. We have

$$Q_{\min}=\sum_{i=1}^{N}\sqrt{P_{1}P_{2}}\sqrt{\alpha_{i}\beta_{i}}s_{ii^{\prime }}=\sqrt{P_{1}P_{2}}\sum_{i=1}^{N}\sqrt{\alpha_{i}\beta_{i}}s_{ii^{\prime }}=\sqrt{P_{1}P_{2}}s^{*}=Q_{\min}^{\prime }.$$

Case (ii): $\sqrt{\frac{P_{1}\alpha_{i}}{P_{2}\beta_{i}}}<s_{ii^{\prime }}<1$, which gives rise to $s^{*}>\sqrt{\frac{P_{1}\beta_{i}}{P_{2}\alpha_{i}}}$. All of the vectors included in $\rho_{1}$ are neglected in the optimal solution for discrimination of $\rho_{1}$ and $\rho_{2}$. According to the Cauchy–Schwarz inequality, the optimal failure probability for succeeding in discriminating $|\Psi_{1}\rangle$ from $|\Psi_{2}\rangle$ satisfies

(35) $$\begin{array}{ccc} Q_{\min}^{\prime } & = & P_{1}+P_{2}{\left(\sum_{i=1}^{N}\sqrt{\alpha_{i}\beta_{i}}s_{ii^{\prime }}\right)}^{2}\leq P_{1}+P_{2}\left(\sum_{j=1}^{N}\alpha_{i}\right)\left(\sum_{i=1}^{N}\beta_{i}\overset{2}{\underset{ii^{\prime }}{s}}\right)\\ & = & P_{1}+P_{2}\left(\sum_{i=1}^{N}\beta_{i}s_{ii^{\prime }}^{2}\right)=Q_{\min}. \end{array}$$

This upper bound is saturated when

$$\frac{\alpha_{1}}{\beta_{1}s_{11^{\prime }}^{2}}=\frac{\alpha_{2}}{\beta_{2}s_{22^{\prime }}^{2}}=...=\frac{\alpha_{N}}{\beta_{N}s_{NN^{\prime }}^{2}}.$$

Case (iii): $0<s_{ii^{\prime }}\leq \sqrt{\frac{P_{1}\alpha_{i}}{P_{2}\beta_{i}}}$ (1≤i≤m), $\sqrt{\frac{P_{1}\alpha_{i}}{P_{2}\beta_{i}}}<s_{ii^{\prime }}\leq 1$ (m+1≤i≤N) and $s^{*}\leq \sqrt{\frac{P_{1}}{P_{2}}}$, where 1<m<N.

The difference ΔQ between the two schemes is given by

(36) $$\begin{array}{ccc} \Delta Q & = & Q_{\min}-Q_{\min}^{\prime }\\ & = & \sum_{i=1}^{m}2\sqrt{P_{1}P_{2}\alpha_{i}\beta_{i}}s_{ii^{\prime }}\;+\;\sum_{i=m+1}^{N}\left(P_{1}\alpha_{i}\;+\;P_{2}\beta_{i}\overset{2}{\underset{ii^{\prime }}{s}}\right)\;-\;2\sqrt{P_{1}P_{2}}\sum_{i=1}^{N}\sqrt{\alpha_{i}\beta_{i}}s_{ii^{\prime }}\\ & = & \sum_{i=m+1}^{N}{\left(\sqrt{P_{1}\alpha_{i}}-\sqrt{P_{2}\beta_{i}}s_{ii^{\prime }}\right)}^{2}>0. \end{array}$$

Case (iv): $0<s_{ii^{\prime }}\leq \sqrt{\frac{P_{1}\alpha_{i}}{P_{2}\beta_{i}}}$ (1≤i≤m), $\sqrt{\frac{P_{1}\alpha_{i}}{P_{2}\beta_{i}}}<s_{ii^{\prime }}\leq 1$ (m+1≤i≤N) and $s^{*}>\sqrt{\frac{P_{1}}{P_{2}}}$. We have

(37) $$\Delta Q=\sum_{i=1}^{m}2\sqrt{P_{1}P_{2}\alpha_{i}\beta_{i}}s_{ii^{\prime }}\;+\;\sum_{i=m+1}^{N}\left(P_{1}\alpha_{i}\;+\;P_{2}\beta_{i}s_{ii^{\prime }}^{2}\right)\;\;-\;P_{1}\;-\;P_{2}{\left(\sum_{i=1}^{N}\sqrt{\alpha_{i}\beta_{i}}s_{ii^{\prime }}\right)}^{2}.$$

We prove ΔQ≥0 via mathematical induction. First, consider the case for m=1. We have

(38) $$\Delta Q_{1}=\sqrt{P_{1}P_{2}\alpha_{1}\beta_{1}}s_{11^{\prime }}\;+\;\sum_{i=2}^{N}\left(P_{1}\alpha_{i}\;+\;P_{2}\beta_{i}s_{ii^{\prime }}^{2}\right)\;-P_{1}-P_{2}s^{*2}.$$

This expression is a quadric function of the variable $s_{11^{\prime }}$ with a negative quadratic coefficient $-P_{2}\alpha_{1}\beta_{1}$. Since $0<s_{11^{\prime }}\leq \sqrt{\frac{P_{1}\alpha_{1}}{P_{2}\beta_{1}}}$, ΔQ achieves its minimum (lower limit) at the boundary points $s_{11^{\prime }}\to 0$ and $s_{11^{\prime }}=\sqrt{\frac{P_{1}\alpha_{1}}{P_{2}\beta_{1}}}$.

Then, according to Equation (38), $s^{*}>\sqrt{\frac{P_{1}}{P_{2}}}$, $s_{11^{\prime }}\to 0$, $\sum_{i=1}^{N}\beta_{i}=1$ and the Cauchy–Schwarz inequality, we have

(39) $$\begin{array}{ccc} \Delta Q_{1} & = & \sum_{i=2}^{N}P_{1}\alpha_{i}\;+\;\frac{P_{2}\sum_{i=2}^{N}\alpha_{i}\sum_{i=2}^{N}\beta_{i}\overset{2}{\underset{ii^{\prime }}{s}}}{1-\alpha_{1}}\;-\;P_{1}\;-\;P_{2}s^{*2}\\ & \geq & P_{1}\left(1\;-\;\alpha_{1}\right)\;+\;P_{2}\left(\frac{1}{1\;-\;\alpha_{1}}\;-\;1\right)\sum_{i=2}^{N}\sqrt{\alpha_{i}\beta_{i}}s_{ii^{\prime }}\;-\;P_{1}\\ & > & -P_{1}\alpha_{1}+\frac{P_{2}\alpha_{1}}{1-\alpha_{1}}\sqrt{\frac{P_{1}}{P_{2}}}>-P_{1}\alpha_{1}+\frac{P_{2}\alpha_{1}}{1-\alpha_{1}}\frac{P_{1}}{P_{2}}=\frac{P_{1}\alpha_{1}^{2}}{1-\alpha_{1}}>0. \end{array}$$

Corresponding to another boundary point $s_{11^{\prime }}=\sqrt{\frac{P_{1}\alpha_{1}}{P_{2}\beta_{1}}}$, we have

(40) $$\begin{array}{ccc} \Delta Q_{1} & = & 2\sqrt{\frac{P_{1}\alpha_{1}}{P_{2}\beta_{1}}}\sqrt{P_{1}\alpha_{1}P_{2}\beta_{1}}+\sum_{i=2}^{N}\left(P_{1}\alpha_{i}+P_{2}\beta_{i}\overset{2}{\underset{ii^{\prime }}{s}}\right)-P_{1}-P_{2}{\left(\sum_{i=1}^{N}\sqrt{\alpha_{i}\beta_{i}}s_{ii^{\prime }}\right)}^{2}\\ & = & 2P_{1}\alpha_{1}+P_{1}\left(1-\alpha_{1}\right)+\frac{P_{2}\sum_{i=2}^{N}\alpha_{i}\sum_{i=2}^{N}\beta_{i}\overset{2}{\underset{ii^{\prime }}{s}}}{1-\alpha_{1}}-P_{1}-P_{2}{\left(\sqrt{\frac{P_{1}}{P_{2}}}\alpha_{1}+\sum_{i=2}^{N}\sqrt{\alpha_{i}\beta_{i}}s_{ii^{\prime }}\right)}^{2}\\ & \geq & P_{1}\alpha_{1}+\frac{P_{2}}{1-\alpha_{1}}{\left(\sum_{i=2}^{N}\sqrt{\alpha_{i}\beta_{i}}s_{ii^{\prime }}\right)}^{2}-P_{2}{\left(\sqrt{\frac{P_{1}}{P_{2}}}\alpha_{1}+\sum_{i=2}^{N}\sqrt{\alpha_{i}\beta_{i}}s_{ii^{\prime }}\right)}^{2}\\ & = & \frac{\alpha_{1}}{1-\alpha_{1}}{\left[\sqrt{P_{1}}\left(1-\alpha_{1}\right)-\sqrt{P_{2}}\sum_{i=2}^{N}\sqrt{\alpha_{i}\beta_{i}}s_{ii^{\prime }}\right]}^{2}\geq 0. \end{array}$$

As an induction hypothesis, we suppose that our conclusion holds for m=k, 1<k<N,

(41) $$\Delta Q_{k}=T+2\sqrt{P_{1}\alpha_{k}P_{2}\beta_{k}}s_{kk^{\prime }}+P_{1}\alpha_{k+1}+P_{2}\beta_{k+1}s_{k+1,{\left(k+1\right)}^{\prime }}^{2}+M-P_{1}-P_{2}{\left(\sum_{i=1}^{N}\sqrt{\alpha_{i}\beta_{i}}s_{ii^{\prime }}\right)}^{2}>0,$$

where

$$T=2\sqrt{P_{1}P_{2}}\left(\sum_{i=1}^{k-1}\sqrt{\alpha_{i}\beta_{i}}s_{ii^{\prime }}\right),\;\;\;M=\sum_{i=k+2}^{N}\left(P_{1}\alpha_{i}+P_{2}\beta_{i}s_{ii^{\prime }}^{2}\right).$$

Then, for m=k+1, we have

$$\Delta Q_{k+1}=T+2\sqrt{P_{1}\alpha_{k}P_{2}\beta_{k}}s_{kk^{\prime }}+2\sqrt{P_{1}\alpha_{k+1}\beta_{k+1}}s_{{\left(k+1\right)}^{\prime },k+1}+M-P_{1}-P_{2}{\left(\sum_{i=1}^{N}\sqrt{\alpha_{i}\beta_{i}}s_{ii^{\prime }}\right)}^{2}.$$

Here, $\Delta Q_{k+1}$ can also be considered as a quadratic function of *x* ($x=s_{{\left(k+1\right)}^{\prime },k+1}$) with a minus coefficient of quadratic term. Thus, we also can acquire the minimum $\Delta Q_{\min}$ at the boundary points x=0 and $\sqrt{\frac{P_{1}\alpha_{k+1}}{P_{2}\beta_{k+1}}}$. For x=0, according to that $s^{*}>\sqrt{\frac{P_{1}}{P_{2}}}$, we have

(42) $$\begin{array}{ccc} & & \Delta Q_{k+1}{|}_{x=0}-\Delta Q_{k}{|}_{x=\sqrt{\frac{P_{1}\alpha_{k+1}}{P_{2}\beta_{k+1}}}}\\ & = & P_{1}\alpha_{k+1}^{2}+2\sqrt{P_{1}P_{2}}\left(\;\sum_{i\neq k+1}\sqrt{\alpha_{i}\beta_{i}}s_{ii^{\prime }}\right)\alpha_{k+1}\;-2P_{1}\alpha_{k+1}\\ & = & P_{1}\alpha_{k+1}^{2}+2\sqrt{P_{1}P_{2}}s^{*}\alpha_{k+1}\;-2P_{1}\alpha_{k+1}>P_{1}\alpha_{k+1}^{2}\geq 0. \end{array}$$

Hence, we have

$$\Delta Q_{k+1}{|}_{x=0}>\Delta Q_{k}{|}_{x=\sqrt{\frac{P_{1}\alpha_{k+1}}{P_{2}\beta_{k+1}}}}>0$$

according to the relation (41). For another boundary point $x=\sqrt{\frac{P_{1}\alpha_{k}}{P_{2}\beta_{k}}}$, we have

$$\Delta Q_{k\;+\;1}{|}_{x\;\to \;\sqrt{\frac{P_{1}\alpha_{k}}{P_{2}\beta_{k}}}}\;=\;\Delta Q_{k}{|}_{x\;=\;\sqrt{\frac{P_{1}\alpha_{k}}{P_{2}\beta_{k}}}}>0.$$

Therefore, $\Delta Q_{k+1}>0$. □

**Figure 3:**
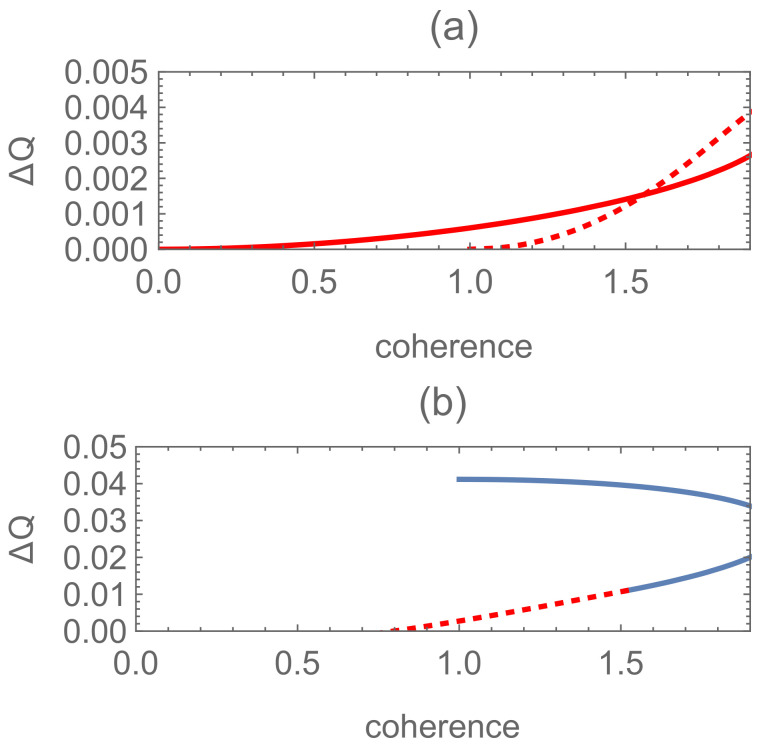
The difference ΔQ of the minimum failure probability between the two schemes as functions of the global coherence $C_{l_{1}}(|\Psi_{i}\rangle )$ (i=1,2) corresponding to the cases for N=3, $P_{1}=0.15$, and $P_{2}=0.85$. (**a**,**b**) correspond to $s_{11^{\prime }}=0.2$, $s_{22^{\prime }}=0.5$, $s_{33^{\prime }}=0.5$ and $s_{11^{\prime }}=0.5$, $s_{22^{\prime }}=0.2$, and $s_{33^{\prime }}=0.2$, respectively. The solid and dotted line corresponds to case (ii) and (iii) in Theorem 3, respectively.

Hence, it can be concluded that the discrimination of the pure superposed states is bound to be more possible to succeed than the mixed ones due to the equal-fidelity condition (31). Namely, the results of Theorem 1 in [22] can be generalized to this rank-*N* system successfully. Set N=3, $\beta_{1}=\alpha_{1}$ and $\alpha_{i}=\beta_{i}=\frac{1-\alpha_{1}}{2}$ (i=2,3). Here, different from filtering, coherence exists symmetrically in the two pure states $|\Phi_{1}\rangle$ and $|\Phi_{2}\rangle$. Then, let us consider the difference $\Delta Q=Q_{\min}-\overset{\prime }{\underset{\min}{Q}}$ as a function of the global coherence measured by the $l_{1}$ norm. It shows that there are much more non-vanishing and helpful coherence regions in which ΔQ>0 than that for quantum filtering problems, see Figure 3. The superiority of pure-pure scheme is inferior to the results of quantum filtering obviously.

## 4. Infinite-Dimensional Systems

Now, we aim to extend the above state discrimination problems to infinite-dimensional systems associated with two Gaussian states in quantum optics. First, we consider the following examples including the results for binomial states [35] as an intermediate transition from finite to infinite-dimensional system problems.

**Example** **1.** *Equal-fidelity cases for comparison of pure-mixed and pure-pure state discrimination.*

In this example, we discriminate a pure state $|\Psi_{1}\rangle$ from one of the following two states:

(1) a rank-*N* mixed state with the eigenvalues corresponding to the binomial distribution [35], which is equivalent to the expression of the Poisson distribution for N→∞:
  (43) $$\overset{0}{\underset{2}{\rho }}=\sum_{i=0}^{N}|f_{0}{\left(\alpha ,i\right)|}^{2}|\Psi_{i^{\prime }}\rangle \langle \Psi_{i^{\prime }}|,$$
  where
  (44) $$f_{0}\left(\alpha ,i\right)=\sqrt{\frac{N!}{i!(N-i)!}{\left(\frac{\alpha^{2}}{N}\right)}^{i}{\left(1-\frac{\alpha^{2}}{N}\right)}^{N-i}};$$
(2) a rank-*∞* mixed state:
  (45) $$\rho_{2}=\sum_{i=0}^{\infty }{\left|f\left(\alpha ,i\right)\right|}^{2}|\Psi_{i^{\prime }}\rangle \langle \Psi_{i^{\prime }}|,$$
  where the function f(α,i) corresponds to the photon number distribution of a given Gaussian state (the well-known coherent or squeezed vacuum state) which is notable in quantum optics. For the case associated with the well-known coherent state, we have
  (46) $$f\left(\alpha ,i\right)=e^{-{\left|\alpha \right|}^{2}/2}\alpha^{i}/\sqrt{i!},$$
  where $\alpha =\left|\alpha \right|e^{i\theta }=re^{i\theta }$; for the one with respect to the squeezed vacuum state, one has
  (47) $$f\left(\alpha ,i\right)=\frac{[-e^{i\theta }{\tanh r(|\alpha |)]}^{i}{\left(2i!\right)}^{1/2}}{\sqrt{\cosh r(|\alpha |)}i!2^{i}}$$
  with
  (48) $$r\left(|\alpha |\right)=\ln(|\alpha |+\sqrt{{\left|\alpha \right|}^{2}+1}).$$

As the binomial distribution of photon numbers is equivalent to Poisson distribution when N→∞, we obtain that

(49) $$\lim_{N\to \infty }\left|f_{0}\left(\alpha ,i\right)\right|=\frac{{\left|\alpha \right|}^{i}}{\sqrt{i}!}e^{-{\left|\alpha \right|}^{2}/2}.$$

From the relation (48), we see that $\sinh^{2}\left[r(|\alpha |)\right]={\left|\alpha \right|}^{2}$, which guarantees the equivalence of average photon number between the generalized coherent and the squeezed vacuum states.

The relations (2) and (28) are also satisfied for both $\rho_{2}^{0}$ and $\rho_{2}$. That is, the vector $|\Psi_{1}\rangle$ is only overlapped with the *t*th vector $|\Psi_{t^{\prime }}\rangle$ in $\rho_{2}$ ($\rho_{2}^{0}$). Then, we discriminate the state $|\Psi_{1}\rangle$ from a superposed state $|\Psi_{2}^{0}\rangle$ and a generalized Gaussian state $|\Psi_{2}\rangle$ given by

(50) $$\begin{array}{ccc} |\Psi_{2}^{0}\rangle & = & \sum_{i=0}^{N}f_{0}\left(\alpha ,i\right)|\Psi_{i^{\prime }}\rangle ; \end{array}$$

(51) $$\begin{array}{ccc} |\Psi_{2}\rangle & = & \sum_{i=0}^{\infty }f\left(\alpha ,i\right)|\Psi_{i^{\prime }}\rangle . \end{array}$$

One can easily obtain that $F(|\Psi_{1}\rangle \langle \Psi_{1}|,\rho_{2}^{0})=F(|\Psi_{1}\rangle ,|\Psi_{2}^{0}\rangle )$ and $F(|\Psi_{1}\rangle \langle \Psi_{1}|,\rho_{2})$$=F(|\Psi_{1}\rangle ,|\Psi_{2}\rangle )$, corresponding to the equal-fidelity condition (28).

**Example** **2.** *Equal-fidelity cases for comparison of mixed-mixed and pure-pure state discrimination.*

In this example, we consider the discrimination of the two pairs of states occurring with prior probabilities $P_{1}$ and $P_{2}$:

(1) rank-*N* mixed states
  (52) $$\rho_{1}^{0}=\sum_{i=0}^{N}|f_{0}{\left(\alpha ,i\right)|}^{2}|\Psi_{i}\rangle \langle \Psi_{i}|,\;\;\;\rho_{2}^{0}=\sum_{i=0}^{N}|f_{0}{\left(\alpha ,i\right)|}^{2}|\Psi_{i^{\prime }}\rangle \langle \Psi_{i^{\prime }}|;$$
(2) rank-*∞* mixed states
  (53) $$\rho_{1}=\sum_{i=0}^{\infty }{\left|f\left(\alpha ,i\right)\right|}^{2}|\Psi_{i}\rangle \langle \Psi_{i}|,\;\;\;\rho_{2}=\sum_{i=0}^{\infty }{\left|f\left(\alpha ,i\right)\right|}^{2}|\Psi_{i^{\prime }}\rangle \langle \Psi_{i^{\prime }}|,$$
  where $|\Psi_{i}\rangle$ and $|\Psi_{i^{\prime }}\rangle$ are orthonormal bases satisfying Equation (31) for 0≤i<∞.

Then, we consider the discrimination of pure states with the two sets of bases superposed as follows:

(54) $$\begin{array}{ccc} |\Phi_{1}^{0}\rangle & \;=\; & \sum_{i=0}^{N}\;f_{0}\left(\alpha ,i\right)\;|\Psi_{i}\rangle ,\;\;\;|\Phi_{2}^{0}\rangle \;=\;\sum_{i=0}^{N}\;f_{0}\left(\alpha ,i\right)\;|\Psi_{i^{\prime }}\rangle ; \end{array}$$

(55) $$\begin{array}{ccc} |\Phi_{1}\rangle & \;=\; & \sum_{i=0}^{\infty }\;f\left(\alpha ,i\right)\;|\Psi_{i}\rangle ,\;\;\;|\Phi_{2}\rangle \;=\;\sum_{i=0}^{\infty }\;f\left(\alpha ,i\right)\;|\Psi_{i^{\prime }}\rangle , \end{array}$$

where $|\Phi_{j}^{0}\rangle$ ($|\Phi_{j}\rangle$) (j=1,2) is a superposed binomial state (generalized Gaussian state) that satisfies $F\left(\rho_{1}^{0},\rho_{2}^{0}\right)=F(|\Phi_{1}^{0}\rangle ,|\Phi_{2}^{0}\rangle )$ ($F\left(\rho_{1},\rho_{2}\right)=F(|\Phi_{1}\rangle ,|\Phi_{2}\rangle )$) obviously.

Concerning the role played by the coherence in the above two examples, we choose the relative entropy coherence [30,34] defined by Equation (15), since the $l_{1}$-norm coherence does not fulfill that the coherence is finite for N→∞. Calculating the global coherence of $|\Phi_{j}^{0}\rangle$ and $|\Phi_{j}\rangle$ (j=1,2) measured by the relative entropy, we have

(56) $$C_{\mathrm{rel}}=-\sum_{i=0}^{N}|f_{0}{\left(\alpha ,i\right)|}^{2}\log|f_{0}{\left(\alpha ,i\right)|}^{2};\;\;\;C_{\mathrm{rel}}=-\sum_{i=0}^{\infty }{\left|f\left(\alpha ,i\right)\right|}^{2}\log{\left|f\left(\alpha ,i\right)\right|}^{2}.$$

**Figure 4:**
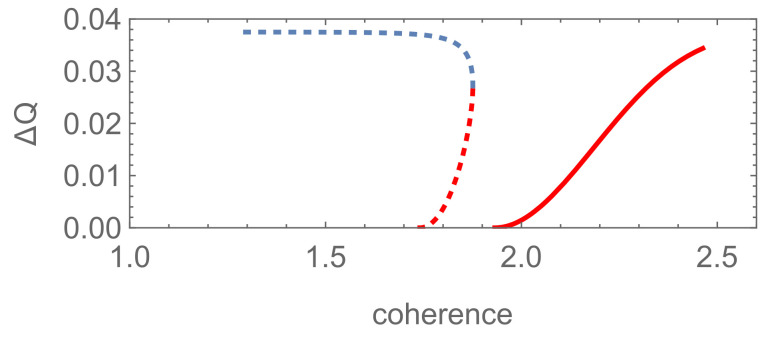
Results for Example 1 (a): the difference ΔQ of the optimal success probabilities between the pure-mixed ($|\Psi_{1}\rangle$ and $\rho_{2}^{0}$) and pure-pure state ($|\Psi_{1}\rangle$ and $|\Psi_{2}^{0}\rangle$) discrimination as a function of the coherence for the finite dimensional state $|\Psi_{2}^{0}\rangle$ with binomial distributed probability amplitude. Dotted line for N=10 and solid line for N=100, where $P_{1}=0.15$, t=0 and $s_{1t^{\prime }}=0.5$.

**Figure 5:**
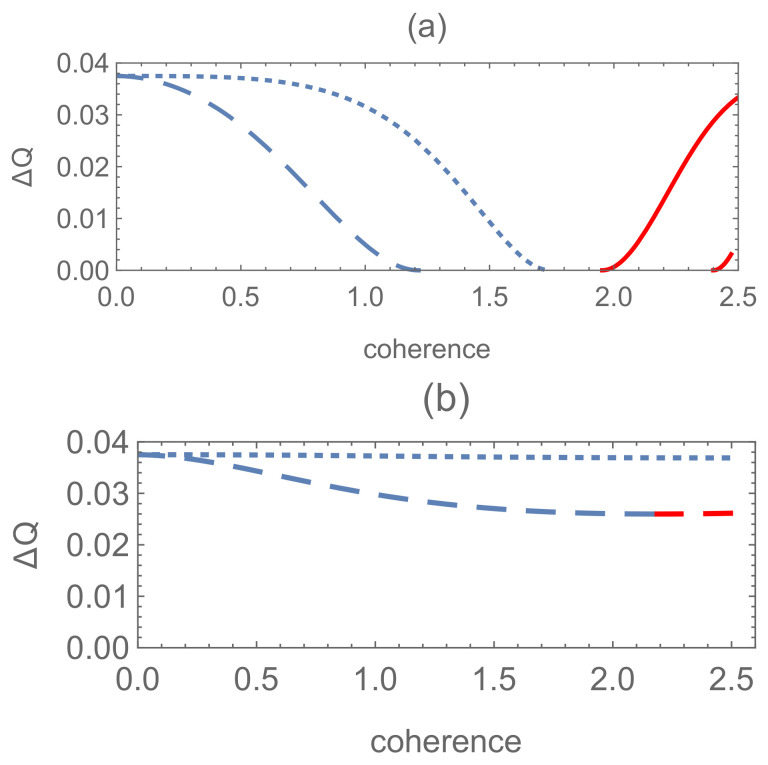
Results for Example 1 (b): the difference ΔQ of the optimal success probabilities between the pure-mixed ($|\Psi_{1}\rangle$ and $\rho_{2}$) and pure-pure ($|\Psi_{1}\rangle$ and $|\Psi_{2}\rangle$) state discrimination as a function of the coherence for the infinite dimensional state $|\Psi_{2}\rangle$, with $P_{1}=0.15$ and $s_{1t^{\prime }}=0.5$. (**a**,**b**) correspond to the scheme with generalized well-known coherent and squeezed vacuum states, respectively. Solid line: t=0; dashed line: t=3; dotted line: t=5.

**Figure 6:**
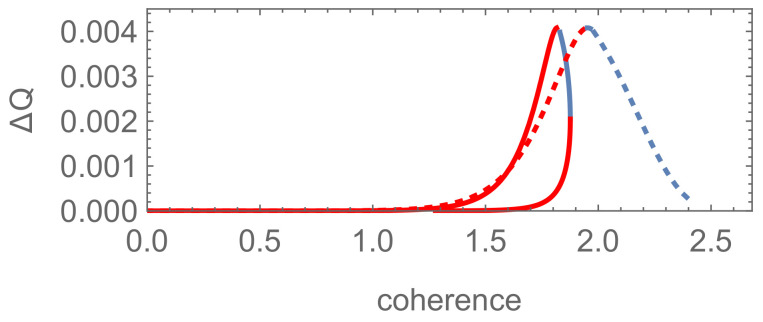
Results for Example 2 (a): the difference ΔQ of the optimal success probabilities between the mixed-mixed ($\rho_{1}^{0}$ and $\rho_{2}^{0}$) and pure-pure ($|\Psi_{1}^{0}\rangle$ and $|\Psi_{2}^{0}\rangle$) state discrimination as a function of the coherence of $|\Psi_{j}^{0}\rangle$ (j=1,2) with $P_{1}=0.15$. Solid line: N=10, $s_{ii^{\prime }}=0.5>\sqrt{\frac{P_{1}}{P_{2}}}$ (i=1,...,4), $s_{ii^{\prime }}=0.2\leq \sqrt{\frac{P_{1}}{P_{2}}}$ (i=5,...,10); dotted line: N=50, $s_{ii^{\prime }}=0.5>\sqrt{\frac{P_{1}}{P_{2}}}$ (i=1,...,4), $s_{ii^{\prime }}=0.2\leq \sqrt{\frac{P_{1}}{P_{2}}}$ (i=5,...,50).

**Figure 7:**
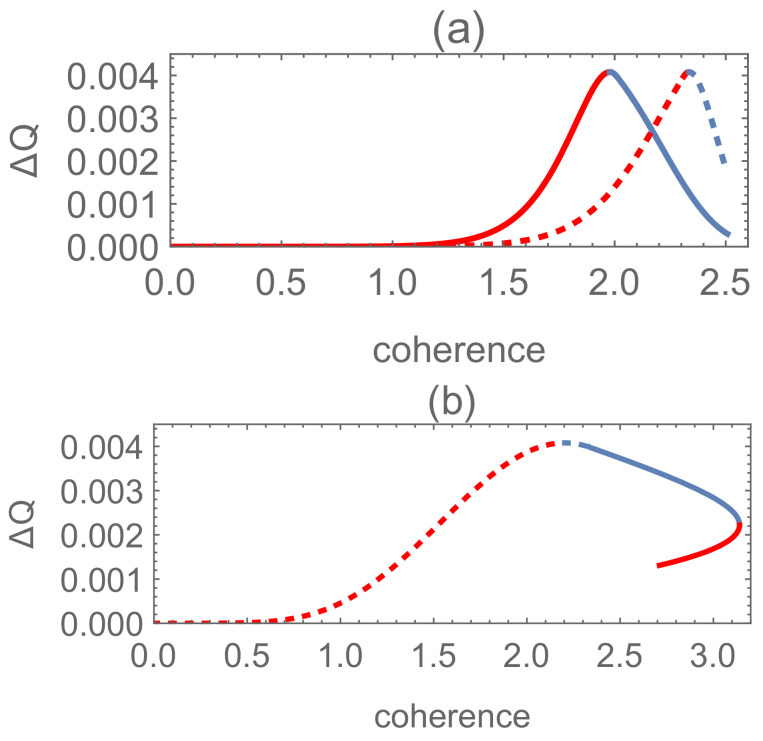
Results for Example 2 (b): the difference ΔQ of the optimal success probabilities between the mixed-mixed ($\rho_{1}$ and $\rho_{2}$) and pure-pure ($|\Psi_{1}\rangle$ and $|\Psi_{2}\rangle$) state discrimination as functions of the coherence $C_{\mathrm{re}}(|\Psi_{i}\rangle )$. (**a**,**b**) correspond to the scheme with generalized well-known coherent and squeezed vacuum states, respectively, for $P_{1}=0.15$ and $P_{2}=0.85$. Solid line: $s_{ii^{\prime }}=0.5>\sqrt{\frac{P_{1}}{P_{2}}}$ (i=1,...,4), $s_{ii^{\prime }}=0.2\leq \sqrt{\frac{P_{1}}{P_{2}}}$ (i=5,...,∞); dotted line: $s_{ii^{\prime }}=0.2$ (i=1,...,4), $s_{ii^{\prime }}=0.5$ (i=5,...,∞).

For the two examples above, the difference ΔQ of the optimal success probabilities between the pure (mixed)-mixed and pure-pure state discrimination is presented in Figure 4, Figure 5, Figure 6 and Figure 7, corresponding to different schemes respectively. It shows that the pure-pure state discrimination scheme is also superior to the pure (mixed)-mixed one and the coherence which is detrimental and helpful to state discriminations coexists irrespective of any schemes involved in the above two examples.

For the quantum filtering including finite-dimensional systems associated with the binomial distribution of photon numbers (shown in Figure 4), the quantum coherence which is helpful to state discriminations can be acquired for larger *N*, which is not the case for the quantum filtering scheme in Figure 2. Then, we make an extension to infinite-dimensional systems corresponding to the generalized Gaussian states in Figure 5. As N→∞, the results of quantum filtering corresponding to the mixed states (43) and (45) gives rise to the same results due to the relation (49). In addition, from the results in Figure 5, it is indicated that as the parameter *t* increases, the helpful coherence encoded in the well-known coherent state decreases by the contrary. While for the scheme with the generalized squeezed vacuum state, despite the superiority of the pure-pure state scheme versus the pure-mixed one, the coherence contributes very little to this superiority, as is shown in Figure 5b.

For the results corresponding to mixed-mixed state discrimination schemes shown in Figure 6 and Figure 7, compared with the results of quantum filtering in Figure 4 and Figure 5, it can be concluded that the symmetrically (asymmetrically) distributed coherence is always helpful (detrimental) to state discrimination for lower dimensional systems. As the dimension increases, symmetrically (asymmetrically) distributed coherence may become detrimental (helpful) on the contrary. For N→∞, just like quantum filtering, the result for the binomial state is also equivalent to the one of the well-known coherent states for this mixed-mixed state discrimination scheme.

We also see that only a small range of helpful coherence is vital for state discrimination, while the others have little effect for the schemes with high-dimensional binomial and the generalized well-known coherent states, as shown in Figure 6 and Figure 7a (solid line). In the cases including the generalized squeezed vacuum states, Figure 7b shows that there are more regions of helpful coherence for the mixed-mixed scheme. Since the well-known coherent state is the eigenstate of the annihilation operator, it saturates the lower bound of the quantum uncertainty relation for momentum and position exactly (ΔpΔq=ℏ/2). That is, the well-known coherent state approaches the boundary between classical and quantum physics. Just because of this property, the coherence encoded in the infinite-dimensional systems associated with this well-known state exhibits so many abnormal behaviors in unambiguous state discrimination, different from the results for both finite-dimensional systems and infinite ones associated with the squeezed vacuum states.

Concerning the related experiments in quantum optics, the discrimination of infinite dimensional quantum states such as the well-known coherent states is a subject of research significance [36,37,38,39,40]. The phases in the well-known coherent state $|\alpha \rangle =e^{-{\left|\alpha \right|}^{2}/2}\sum_{i=0}^{\infty }\alpha^{i}/\sqrt{i!}|i\rangle$ ($\alpha =\left|\alpha \right|e^{i\theta }$) are randomized under quantum decoherence. Then taking the average over the variable θ, one has

$$\frac{1}{2\pi }\int_{0}^{2\pi }|\alpha \rangle \langle \alpha |d\theta =\sum_{i=0}^{\infty }\frac{1}{i!}{\left|\alpha \right|}^{2i}|i\rangle \langle i|.$$

Hence, the mixed state in (45) can be prepared successfully. Otherwise, the state can also be acquired via local measurements on a two-mode well-known coherent state.

## 5. Conclusions

We have investigated the discrimination between a pure state and a rank-*N* mixed state (quantum filtering) and compared its optimal successful probability with the one for discriminating another two pure states. One state involved in the pure-pure scheme is identical to the one in quantum filtering; the other one is superposed by the eigenvectors of the above-mentioned mixed state. As the pure-mixed and pure-pure states have the same fidelity, we prove that the optimal success probability of a pure-pure state scheme is superior to quantum filtering. For lower dimensional systems, e.g., N=2,3, the coherence encoded in the pure state is detrimental to state discrimination. If the equal-fidelity restriction is relaxed and the phases in the constructed coherent pure states are identical to each other, the superiority of the pure-pure state scheme is impaired severely. As we adjust the phases to proper values, the superiority of the pure-pure scheme revives, and helpful coherence is acquired. However, this superiority emerges not surprisingly because of a lower fidelity between the two pure states versus the pure-mixed one.

After discriminating two rank-*N* (*N* is a finite positive integer) mixed states whose eigenvectors have one-to-one non-zero overlaps (mixed-mixed state scheme), we also consider the discrimination of two pure states which are superposed by the eigenvectors. Thus, the pure-pure and mixed-mixed states also have the same fidelity. We also prove that the pure-pure state scheme is bound to be superior to the mixed-mixed one. Namely, the result of *Theorem 1* in Ref. [22] confined to rank-two systems is generalized to rank-*N* systems successfully. Due to the symmetrical distribution of coherence encoded in the two pure superposed states, different from the result of quantum filtering, the coherence is always helpful to state discrimination for lower-dimensional systems.

Finally, in order to generalize our results to infinite-dimensional systems, we have first considered the examples of discriminating binomial states. For higher dimensional systems, we remark that some asymmetrically (symmetrically) distributed coherence which is helpful (detrimental) to state discrimination occurs, which turns to be more apparent after we made an extension to infinite-dimensional systems (N→∞) associated with the well-known coherent rather than squeezed-vacuum states. These results can be attributed to the fact that the well-known coherent state which saturates the lower bound of the quantum uncertainty relation for momentum and position approaches the boundary between classical and quantum physics.

Sequential state discrimination (SSD) provided in [13] is a scheme for discriminating one sender’s quantum states via *N* observers who are separately located. SSD is investigated sequentially in [16,17,22,41]. As a next step, we plan to investigate another interesting problem corresponding to SSD including quantum filtering and rank-*N* mixed states discriminations and consider the role played by quantum correlation and coherence in the procedure.

## Data Availability

Data sharing not applicable.
